# The time of onset of intradialytic hypotension during a hemodialysis session associates with clinical parameters and mortality

**DOI:** 10.1016/j.kint.2021.01.018

**Published:** 2021-06

**Authors:** David F. Keane, Jochen G. Raimann, Hanjie Zhang, Joanna Willetts, Stephan Thijssen, Peter Kotanko

**Affiliations:** 1 Research Division, Renal Research Institute, New York, New York, USA; 2 Medical Physics and Engineering, Leeds Teaching Hospitals NHS Trust, Leeds, UK; 3 Leeds Institute for Cardiovascular and Metabolic Medicine, University of Leeds, Leeds, UK; 4 Fresenius Medical Care, Global Medical Office, Waltham, Massachusetts, USA; 5 Division of Nephrology, Icahn School of Medicine at Mount Sinai, New York, New York, USA

**Keywords:** blood volume, hemodialysis, intradialytic hypotension, oximetry, survival

## Abstract

Intradialytic hypotension (IDH) is a common complication of hemodialysis, but there is no data about the time of onset during treatment. Here we describe the incidence of IDH throughout hemodialysis and associations of time of hypotension with clinical parameters and survival by analyzing data from 21 dialysis clinics in the United States to include 785682 treatments from 4348 patients. IDH was defined as a systolic blood pressure of 90 mmHg or under while IDH incidence was calculated in 30-minute intervals throughout the hemodialysis session. Associations of time of IDH with clinical and treatment parameters were explored using logistic regression and with survival using Cox-regression. Sensitivity analysis considered further IDH definitions. IDH occurred in 12% of sessions at a median time interval of 120-149 minutes. There was no notable change in IDH incidence across hemodialysis intervals (range: 2.6-3.2 episodes per 100 session-intervals). Relative blood volume and ultrafiltration volume did not notably associate with IDH in the first 90 minutes but did thereafter. Associations between central venous but not arterial oxygen saturation and IDH were present throughout hemodialysis. Patients prone to IDH early as compared to late in a session had worse survival. Sensitivity analyses suggested IDH definition affects time of onset but other analyses were comparable. Thus, our study highlights the incidence of IDH during the early part of hemodialysis which, when compared to later episodes, associates with clinical parameters and mortality.

## Graphical abstract

**Figure:**
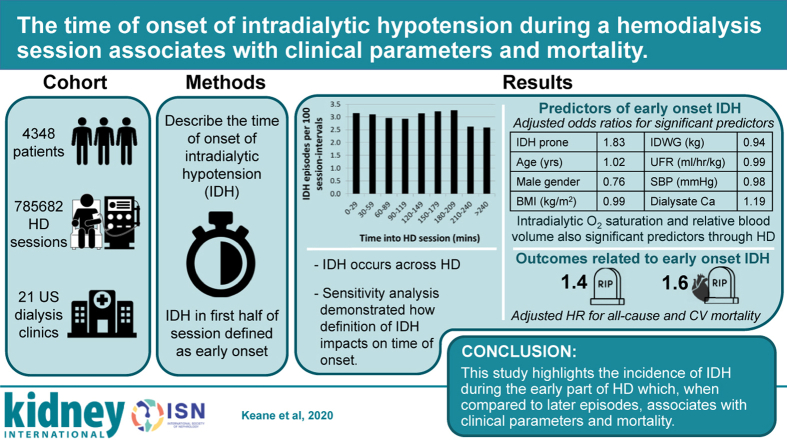

Intradialytic hypotension (IDH) associates with adverse outcomes in hemodialysis (HD) patients, including patient self-reported symptom burden, access failure, cardiovascular events, and mortality.^1^, ^2^, ^3^ Despite being one of the most common complications associated with HD, there is no consensus on the definition, means to prevention, and management of IDH.

The challenges in defining IDH have been reviewed elsewhere,^4^ but generally definitions include some or all of the following: (i) a systolic blood pressure (SBP) nadir; (ii) an absolute reduction in SBP; and (iii) required interventions. Basic physiology suggests that reductions in blood pressure during HD can be related to a combination of volume reduction, impaired cardiac output response to fluid removal, and changes in total peripheral resistance.^5^ Unfortunately, it is not trivial to comprehensively monitor these parameters, so studies are scarce and include small numbers of patients.^6^

Interventions for managing IDH are lacking. There is increasing evidence that use of cooled dialysate can prevent IDH^7^ but as yet is not universally adopted. Other interventions, including use of relative blood volume (RBV),^8^ biofeedback-controlled ultrafiltration rate (UFR), and/or dialysate sodium^9^ and pharmacological management,^10^ all lack a sufficient evidence base for widespread acceptance.

Numerous factors have been shown to associate with IDH occurrence. These include demographic factors, comorbidities, treatment prescriptions, serum osmolality, antihypertensive medications, and anemia.^11^, ^12^, ^13^ RBV at the time of IDH has been shown to have significant intersubject variability, but for a given individual, there appears to be a relatively stable critical RBV preceding IDH.^14^ There are some data to suggest that central venous oxygen saturation (ScvO_2_) decreases more in sessions where IDH occurs.^15^

Studies investigating IDH frequently define sessions based on whether an IDH event occurs, or define patients as being prone to IDH or not, but little attention has been given to how the incidence of IDH onset varies *within* the HD session. We aimed to: (i) characterize the incidence of IDH in discrete time intervals within an HD session; (ii) investigate whether any routinely collected parameters were associated with the time of IDH; and (iii) consider whether the time of IDH is associated with mortality.

## Methods

### Study design

This was a retrospective, observational cohort study utilizing routinely collected treatment data from the network of Renal Research Institute clinics across the United States. We extracted data from all dialysis sessions in 21 clinics between January 1, 2017 and October 31, 2019 from both incident and prevalent patients, with no exclusion criteria. Parameters extracted included demographic and laboratory variables, comorbidities, HD prescriptions, and blood pressure, as well as hematocrit, RBV, and oxygen saturation (SO_2_), measured by the Crit-Line monitor (CLM; Fresenius Medical Care North America, Waltham, MA). The number of patients and sessions in these clinics during the study period determined the sample size. The study was deemed to be exempt from review by the Western Institutional Review Board. To comply with the US Health Insurance Portability and Accountability Act definition of a deidentified data set, patient age was capped at 90 years and dates were transformed to a relative timeline. The study was reported in accordance with Strengthening the Reporting of Observational Studies in Epidemiology guidance.^16^

### Definitions of data analyzed

#### IDH

An intradialytic SBP of <90 mm Hg has been shown to have the greatest association with mortality,^17^ and we used this as our primary definition of IDH. Acknowledging the lack of consensus on IDH definition, we undertook sensitivity analyses with 3 other definitions of IDH. Assimon and Flythe^4^ describe 3 main components of most definitions: characteristics of blood pressure behavior (largely a decline and/or a nadir value), interventions administered, and patient-reported symptoms. Furthermore, it is highlighted that definitions based on a nadir value only are likely to include events that relate to chronic hypotension, which will skew results to earlier times. Our data set had no information on patient-reported symptoms, so we aimed to cover the other components using 3 additional definitions:

(i) A decline in SBP >30 mm Hg from predialysis levels.
(ii) A combination of a nadir SBP <90 mm Hg plus a decline in SBP >30 mm Hg from predialysis levels.
(iii) Nursing intervention based on fluids administered during dialysis.

For the definition based on fluids administered, we removed the final 5 minutes of treatment data to avoid inclusion of rinsing fluids, calculated the modal value for fluids administered per session at each center (assumed to be the priming fluid), and defined IDH as any administered fluid of at least 50 ml once the total fluid administered surpassed the mode for the given center.

Individual subjects were defined as IDH-prone when an IDH episode occurred in >30% of their sessions. For secondary analyses, we also stratified all patients and sessions based on the timing of IDH. All patients with at least one IDH event were classified as prone to early-onset IDH if more than half of recorded IDH episodes occurred before 120 minutes and late onset otherwise. All HD sessions where an IDH session occurred were classified as early or late onset based on whether the first IDH episode in the session was before or after 120 minutes.

#### Clinical and demographic data

Blood pressure measurements were made pre-HD and post-HD, every 30 minutes during the treatment, and when clinically indicated, using automated oscillometric devices connected to the dialysis machine. Age and dialysis vintage were taken from the date of the first treatment session in the study period. Comorbidities are defined by clinicians responsible for the patient’s care, and those extracted included presence or absence of chronic heart failure, diabetes mellitus, and peripheral arterial/vascular disease. Antihypertensive medication use was defined as those patients with at least one open antihypertensive prescription in the first 30 days of the study period. Mortality and cause of death data come from the end-stage renal disease death notification form (CMS 2746), and accompanying categories for cause of death were used to define those deaths as cardiovascular related or not.

#### Intradialytic measurements

The CLM measures hematocrit, SO_2_, and RBV every minute. SO_2_ was included as arterial oxygen saturation (SaO_2_) for all sessions where vascular access was via a fistula or graft and as ScvO_2_ for all sessions utilizing a central venous catheter. Where measurement of ScvO_2_ was available, we also calculated the estimated upper-body blood flow (eUBBF) in L/min throughout the treatment. This was based on the following formula:

$$\text{eUBBF}={\text{SaO}}_{2}-\left[\left(100\text{∗oxygen consumption}\right)/\left(\text{K∗hemoglobin∗}{\text{ScvO}}_{2}\right)\right]$$

where SaO_2_ was assumed to be 92%, ScvO_2_ was measured by CLM, hemoglobin in g/L was calculated as CLM-measured hematocrit/2.94, K, the amount of oxygen (in ml) bound per g of hemoglobin, was 1.34, and oxygen consumption was calculated using demographic information.^18^ Further details on the assumptions underlying this formula can be found in previous work from this group.^19^

To account for the fact that UFR can vary during a session, particularly in a session where IDH occurs, we defined the UFR for a given session as the mean of all recorded UFRs preceding an IDH episode, where an episode occurred, and the mean of all recorded UFRs in a session where it did not. UFR was normalized to postdialysis body weight and reported as ml/hour per kg.

#### Intradialytic timing data

**Figure 1:**
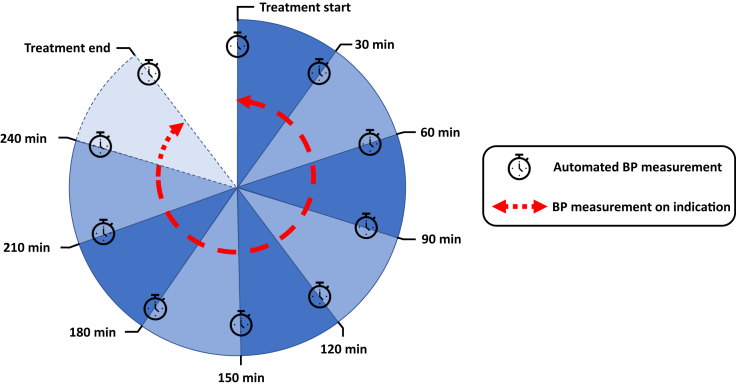
**Hemodialysis sessions-intervals used to define incidence of intradialytic hypotension across a treatment session.** All sessions routinely had automated blood pressure (BP) measured every 30 minutes, whereas BP measured on indication could occur at any point in each session interval.

Given that blood pressure measurements were made every 30 minutes and on indication, it was not possible to define IDH any more accurately than to have occurred within a 30-minute interval. Therefore, we divided each session into 9 intervals, corresponding to 8 30-minute intervals from 0 up to 240 minutes and 1 interval for times exceeding 240 minutes (Figure 1).

### Data analysis

#### Time of IDH onset

The primary objective was to describe the incidence of IDH throughout HD sessions. To calculate the incidence of IDH in any given interval, we divided the total number of IDH events in an interval by the total number of sessions where at least one blood pressure measurement had been made during that interval. Only the first case of IDH in any HD session was included in the analysis. To avoid bias related to subject-level variation in frequency of IDH, we calculated the incidence for each session-interval at the subject level and then averaged across subjects. IDH incidence was reported in each of the 9 intervals as the number of IDH events per 100 sessions-intervals.

#### Intradialytic measurements

Second, we investigated whether hemodynamic parameters measured by CLM and ultrafiltration volume (UFV) were associated with imminent IDH and whether this depended on time into an HD session. We extracted RBV, SO_2_, eUBBF, and UFV at the start of each session-interval and compared parameters between those sessions-intervals where IDH occurred and those where it did not occur in that session-interval. To account for repeated measures on individual subjects, a separate mixed effects regression model was used at each time point with RBV, SO_2_, or UFV as the dependent variable, the presence or absence of IDH as a dummy predictor, and subject as a random effect. This allowed us to compare RBV and SO_2_ for those patients about to experience an IDH episode with those who were stable for at least the following 30 minutes.

#### Associations between time of IDH and clinical variables

To investigate associations between patient, treatment, or HD prescription variables and the time of IDH, we constructed mixed effects logistic regression models, to account for repeated measurements on patients and the possibility of practice pattern differences between centers. Early/late onset of IDH was used as the dependent variable, participant and treatment center were random effects, and the fixed effects were chosen based on plausibility and previous literature. Univariate analysis was performed for all predictor variables, followed by 2 multivariate models: (i) those variables available at all sessions (age, sex, body mass index [BMI], comorbidities, interdialytic weight gain (IDWG), UFR, SBP, dialysis vintage, and dialysate calcium [DCa]); and (ii) those variables from model (i) plus albumin and dialysate to serum sodium gradient, which are only measured one session per month.

#### Survival analysis

We separately considered associations between time of IDH and both all-cause and cardiovascular mortality. We plotted Kaplan-Meier survival curves for patients prone to early-onset IDH and late-onset IDH and generated Cox proportional hazard models for early versus late IDH group, adjusted for age, sex, BMI, chronic heart failure, and diabetes. Patients were censored at the end of the study period, on transfer to a different treatment modality, or out of the group of Renal Research Institute clinics.

#### Statistical analysis

Incidence of IDH was described and summarized using the median session interval for first IDH episode. All regression analyses were based on complete case analysis. Linear regression models were presented graphically as means ± 95% confidence intervals for the 2 dummy-coded conditions representing presence or absence of IDH in the following 30 minutes. Logistic and Cox proportional hazard regression models were presented as odds ratios and 95% confidence intervals. Two-sided *P* values of <0.05 were considered statistically significant. All statistical analyses were performed using R 3.5.0.^20^

## Results

### Study cohort

**Figure 2:**
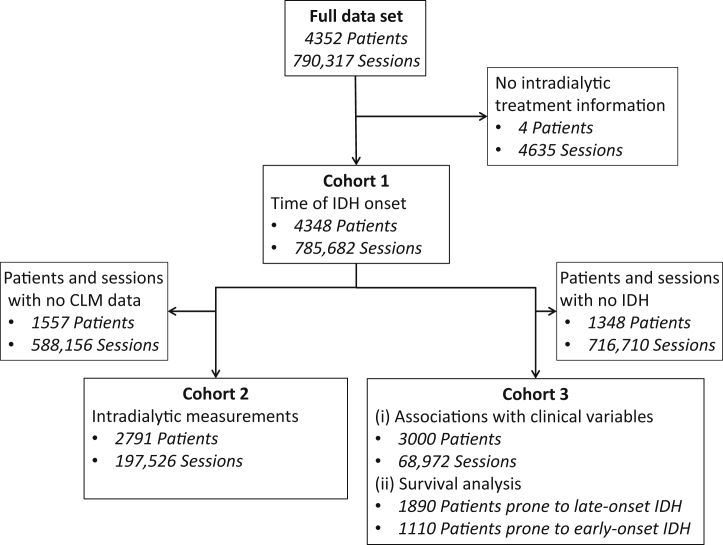
**Patient flow diagram.** CLM, Crit-Line monitor; IDH, intradialytic hypotension.

The extracted data set included 4352 patients with 790,317 sessions from 21 dialysis units. The number of patients and sessions included in each part of the analysis can be seen in Figure 2. Patients prone to IDH were more likely to be older, White, male, and comorbid, have longer dialysis vintage, and less likely to be prescribed an antihypertensive, whereas sessions where IDH occurred had longer treatment times with lower UFR and were linked to higher BMI and lower systolic and diastolic blood pressure and pulse pressure (Table 1).

**Table 1:** Patient and treatment characteristics for the whole cohort and stratified by whether the patient is prone to IDH (>30% sessions) for patient data or whether the session included an IDH episode for session data

Patient data	Total	Not IDH-prone	IDH-prone
No. of patients	4348	3799 (87)	549 (13)
Male sex	2524 (58)	2232 (59)	292 (53)
Dialysis vintage, yr	3.1 (4.1)	2.9 (3.9)	4.7 (5.2)
Age, yr	62.1 (15)	61.7 (15.6)	64.9 (15.0)
Race			
White	1936 (45)	1656 (44)	280 (51)
Black	1475 (34)	1303 (34)	172 (31)
Other	156 (4)	136 (4)	20 (4)
Unknown	781 (18)	704 (19)	77 (14)
Comorbidity			
CHF	423 (10)	335 (9)	88 (16)
Diabetes	978 (23)	826 (22)	152 (28)
PAD-PVD	204 (5)	156 (4)	48 (9)
Antihypertensive use	1054 (24)	969 (26)	85 (15)
Early-onset IDH	1253 (29)	886 (23)	367 (67)

**Session data**	**Total**	**No IDH session**	**IDH session**
No. of sessions	785,682	690,070 (88)	95,612 (12)
BMI, kg/m^2^	28.7 (7.8)	28.5 (7.7)	30.2 (8.9)
IDWG, kg	2.1 (3.0)	2.1 (2.9)	2.2 (3.6)
Treatment time, min	220 (32)	221 (32)	226 (35)
UFR, ml/h per kg	8.8 (9.6)	8.9 (9.1)	7.9 (14.5)
Vascular access			
Fistula/graft	638,337 (81)	563,647 (82)	74,690 (78)
Catheter	147,342 (19)	126,420 (18)	20,922 (22)
Blood flow, ml/min	421 (54)	421 (54)	417 (54)
Pre-HD SBP, mm Hg	149 (27)	152 (27)	128 (23)
Pre-HD DBP, mm Hg	78 (16)	79 (16)	69 (16)
Post-HD SBP, mm Hg	138 (25)	141 (25)	114 (20)
Post-HD DBP, mm Hg	73 (15)	74 (15)	62 (13)
Pulse pressure, mm Hg	71 (21)	72 (21)	59 (21)
Dialysate temperature, °C	36.6 (0.4)	36.6 (0.4)	36.5 (0.5)
Dialysate sodium, mEq/L			
<136	11,735 (1)	10,291 (1)	1444 (2)
136	18,067 (2)	15,670 (2)	2397 (3)
137	732,534 (93)	643,320 (93)	89,214 (93)
138	12,617 (2)	11,254 (2)	1363 (1)
>138	10,729 (1)	9535 (1)	1194 (1)
Dialysate calcium, mEq/L			
2	10,513 (1)	8586 (1)	1924 (2)
2.25	316,381 (40)	276,154 (40)	40,228 (42)
2.5	356,720 (45)	318,235 (46)	38,487 (40)
3	101,379 (13)	86,427 (13)	14,952 (16)
3.5	675 (0.1)	655 (0.1)	20 (0.02)

BMI, body mass index; CHF, chronic heart failure; DBP, diastolic blood pressure; HD, hemodialysis; IDH, intradialytic hypotension; IDWG, interdialytic weight gain; PAD, peripheral artery disease; PVD, peripheral vascular disease; SBP, systolic blood pressure; UFR, ultrafiltration rate.

Data are number (SD) for continuous variables and number (%) for categorical variables.

### Time of IDH onset

**Figure 3:**
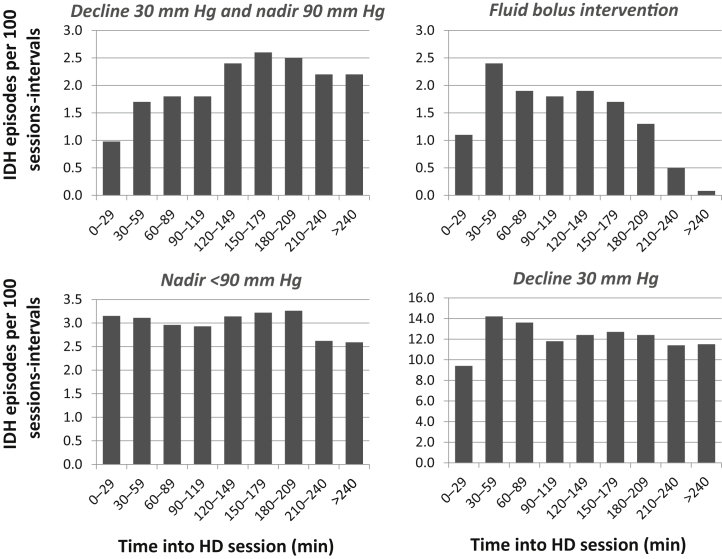
**Intradialytic hypotension (IDH) episodes per 100 sessions-intervals at risk, with time into hemodialysis (HD) session using IDH definitions of decline in systolic blood pressure (SBP) >30 mm Hg and nadir SBP <90 mm Hg; fluids administered; nadir SBP <90 mm Hg; and decline in SBP >30 mm Hg.**

Using our primary definition of IDH (nadir SBP <90 mm Hg), the median session-interval for first IDH in a session was 120 to 149 minutes. There was no clear trend in IDH incidence across session intervals, with a range of 2.6 to 3.2 episodes per 100 sessions-intervals (Figure 3).

### Intradialytic measurements

**Figure 4:**
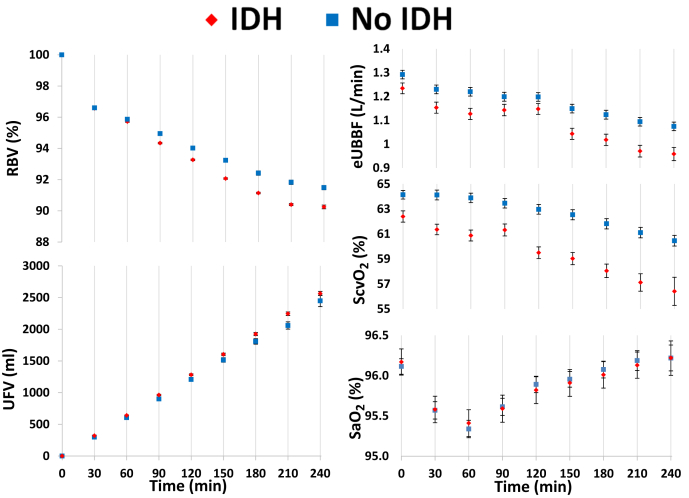
**Intradialytic relative blood volume (RBV), ultrafiltration volume (UFV), arterial oxygen saturation (SaO**_**2**_**), central venous oxygen saturation (ScvO**_**2**_**), and estimated upper-body blood flow (eUBBF).** Each data point represents an average value at the start of every session-interval, stratified by whether intradialytic hypotension (IDH) occurred in the subsequent 30 minutes or not, with error bars representing 95% confidence intervals. Comparisons of each variable at all time points showed statistically significant differences (*P* < 0.05).

Intradialytic RBV, UFV, SO_2_, and eUBBF results are plotted in Figure 4, separated based on whether IDH occurs in the subsequent 30 minutes or not. There were no clinically significant differences in RBV and UFV between sessions-intervals where IDH is imminent compared with those where no episode occurs until 90 minutes, after which RBV and UFV are both notably lower preceding IDH. There was a clinically significant reduction in ScvO_2_ and eUBBF preceding IDH at all time points, whereas no demonstrable difference was observed for SaO_2_.

### Associations between time of IDH onset and clinical variables

Among demographic factors, being prone to IDH, increasing age, female sex, and lower BMI were all associated with early-onset IDH in adjusted models, whereas among treatment factors, higher DCa and lower IDWG, UFR, and SBP showed similar tendencies (Table 2).

**Table 2:** Mixed-effects logistic regression for the odds of IDH occurring in the first half of a session compared with later in the treatment

Variable	Univariate		Multivariate (model 1)		Multivariate (model 2)	
	OR	95% CI	OR	95% CI	OR	95% CI
IDH-prone	2.61	2.35–2.90^a^	1.83	1.67–2.01^a^	1.67	1.43–1.94^a^
Age, yr	1.02	1.01–1.02^a^	1.02	1.01–1.02^a^	1.02	1.01–1.02^a^
Male sex	0.88	0.80–0.96^a^	0.76	0.71–0.82^a^	0.81	0.71–0.94^a^
BMI, kg/m^2^	0.98	0.97–0.98^a^	0.99	0.98–0.99^a^	0.98	0.97–0.99^a^
CHF	1.20	1.05–1.38	1.11	0.99–1.25	1.16	0.95–1.42
Diabetes	0.90	0.81–1.00^a^	0.99	0.91–1.08	1.09	0.93–1.26
PAD-PVD	1.14	0.95–1.38	0.90	0.77–1.05	0.77	0.59–1.00^a^
IDWG, kg	0.90	0.89–0.91^a^	0.94	0.93–0.95^a^	0.90	0.86–0.95^a^
Ultrafiltration rate, ml/h per kg	0.97	0.97–0.98^a^	0.99	0.99–1.00^a^	0.99	0.98–1.01
Pre-SBP, mmHg	0.97	0.97–0.97^a^	0.98	0.97–0.98^a^	0.97	0.97–0.98^a^
Dialysis vintage, yr	1.00	1.00–1.02	1.00	0.99–1.01	1.01	0.99–1.02
Dialysate calcium, mEq/L	1.13	1.02–1.25^a^	1.19	1.08–1.32^a^	1.38	1.07–1.77^a^
Albumin	0.55	0.49–0.61^a^	—	—	0.90	0.76–1.08
Dialysate to serum Na gradient, mEq/L	1.01	0.99–1.03	—	—	1.01	0.99–1.03

BMI, body mass index; CHF, chronic heart failure; CI, confidence interval; IDH, intradialytic hypotension; IDWG, interdialytic weight gain; OR, odds ratio; PAD, peripheral artery disease; PVD, peripheral vascular disease; SBP, systolic blood pressure.

Adjusted analysis for model 1 includes all covariates available for all treatments (785,682 sessions), whereas for model 2, it includes all variables from model 1 plus albumin and sodium gradient in a limited number of treatments due to availability of these data (15,994 sessions).

a *P* < 0.05.

### Survival analysis

**Figure 5:**
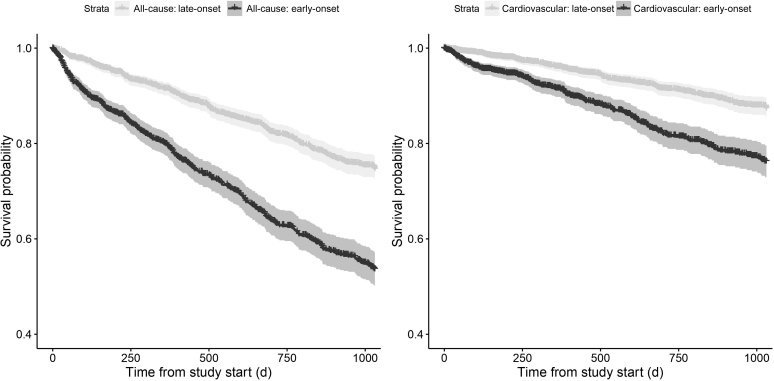
**Kaplan-Meier curves for patients who tended to have intradialytic hypotension (IDH) in the first half of a session compared with those who tended to have IDH in the second half of a session.** Data presented for all-cause and cardiovascular mortality, with accompanying 95% confidence intervals.

During a median follow-up of 628 days, there were 743 deaths, of which 325 were cardiovascular related. Unadjusted Kaplan-Meier analysis showed significantly higher all-cause and cardiovascular mortality for those patients who tended to have IDH early in a session (*P* < 0.001; Figure 5). Adjusted multivariate Cox analysis showed poorer survival in patients prone to early IDH (all-cause mortality hazard ratio, 1.4; 95% confidence interval, 1.2–1.7; cardiovascular mortality hazard ratio, 1.6; 95% confidence interval, 1.2–2.2; Table 3).

**Table 3:** Cox proportional hazards analysis of the association of early-onset IDH with both all-cause and cardiovascular mortality

Variable	All-cause mortality				Cardiovascular mortality			
	Univariate		Multivariate		Univariate		Multivariate	
	OR	95% CI	OR	95% CI	OR	95% CI	OR	95% CI
Early-onset IDH	2.26	1.95–2.61^a^	1.40	1.16–1.69^a^	2.22	1.78–2.78^a^	1.64	1.23–2.18^a^
IDH-prone	2.36	2.00–2.79^a^	1.52	1.22–1.88^a^	1.80	1.37–2.37^a^	1.25	0.88–1.76
Age, yr	1.04	1.04–1.05^a^	1.03	1.03–1.04^a^	1.03	1.02–1.04^a^	1.02	1.01–1.03^a^
Dialysis vintage, yr	1.00	0.99–1.01	1.00	1.00–1.00	1.00	1.00–1.00	1.00	1.00–1.00
Male sex	0.99	0.86–1.15	1.24	1.03–1.48^a^	1.05	0.84–1.32	1.36	1.03–1.80^a^
Race (White vs. non-White)	1.78	1.52–2.09^a^	1.58	1.32–1.90^a^	1.33	1.04–1.69^a^	1.23	0.93–1.61
BMI, kg/m^2^	0.97	0.95–0.98^a^	0.97	0.96–0.99^a^	0.97	0.95–0.98^a^	0.97	0.95–0.99^a^
CHF	1.26	1.03–1.54^a^	1.16	0.93–1.45	1.36	1.01–1.83^a^	1.32	0.95–1.84
Diabetes	1.03	0.88–1.21	0.88	0.73–1.06	1.02	0.80–1.30	0.86	0.65–1.14
Albumin, g/dl	0.25	0.22–0.29^a^	0.27	0.22–0.34	0.31	0.25–0.40^a^	0.35	0.25–0.49^a^
IDWG, kg	0.94	0.91–0.96^a^	1.00	0.07–0.94	0.95	0.91–1.00	1.01	0.96–1.07

BMI, body mass index; CHF, chronic heart failure; CI, confidence interval; IDH, intradialytic hypotension; IDWG, interdialytic weight gain; OR, odds ratio.

a *P* < 0.05.

### Sensitivity analysis

Using a decline-based definition (≥30 mm Hg) of IDH, the incidence was significantly higher than all other definitions (total incidence, 51% of sessions; range of 9.4–14.2 episodes per 100 sessions-intervals), although the median session-interval did not change (Figure 3). Adding a reduction of at least 30 mm Hg from predialysis levels to the definition of IDH significantly reduced the total incidence (9% of sessions) and the incidence of IDH in the early part of HD, with only 1.0 and 1.7 incidents per 100 session intervals in the first and second intervals, compared with 3.2 and 3.1 using our primary definition of IDH (Figure 3). The median session-interval for first IDH increased by 30 minutes, to 150 to 179 minutes. Using fluid administered as a marker of interventions to address IDH produced slightly lower incidence of IDH (9% of total sessions), a wider range of values (0.1–2.4 episodes per 100 sessions-intervals), and a slightly earlier median session-interval of 90 to 119 minutes.

We repeated regression and survival analyses using a combined definition of nadir SBP <90 mm Hg and a decline in SBP ≥30 mm Hg, which produced comparable results to those obtained using the primary defintion (Supplementary Figures S1 and S2 and Supplementary Tables S3 and S4).

## Discussion

We have demonstrated that IDH, defined as SBP <90 mm Hg, occurs throughout HD at a relatively stable rate. This pattern is dependent on how IDH is defined, reinforcing the need for careful consideration of IDH definition in research and clinical management. Furthermore, our results suggest that the ability of RBV and UFV to discriminate imminent IDH onset is dependent on the time into an HD session. Finally, we were able to show differences in clinical and treatment variables as well as survival between patients prone to IDH early and late in a session.

### Time of IDH onset

Despite a general assumption that most IDH episodes occur toward the end of a treatment,^21^ there are little published data on IDH timing. Zucchelli and Santoro described hemodynamic monitoring of IDH-prone patients, showing a mean time of IDH of 178 minutes into a session.^5^ This is notably later than we found, although the study does not have a clear definition of IDH and does not state whether it only includes the first IDH in any given session.

Intradialytic blood pressure changes, which are associated with IDH, have been better characterized. A biphasic reduction in SBP during HD has been described, consisting of a rapid early decline across the first quarter of the treatment and a later, gentler decline, although with significant interindividual variation.^22^^,^^23^ This pattern could be linked to the relatively high incidence of IDH early in a session observed herein.

### Intradialytic measurements

Clearly, the rate of removal of fluid from the circulation by ultrafiltration is central to the pathogenesis of IDH. However, our results show no clinically significant difference in the cumulative UFV at 30, 60, and 90 minutes between sessions where IDH is imminent and where it is not, indicating average UFR was not notably different at these time points. This could potentially point to the other prime driver of fluid out of the circulation: the rapid changes in extracellular osmolality at the start of dialysis, which are associated with osmotic fluid shifts and blood pressure instability.^12^

The use of RBV for prediction and prevention of IDH is well described, but uncertainties around application remain.^24^ The RBV profiles observed herein are consistent with previous data^25^^,^^26^ and mirror the patterns in SBP described above. The novel aspect in these data is that, although differences were statistically significant throughout HD, differences in RBV preceding IDH are so small to be clinically insignificant during the early period of a session. Later in the session, RBV is notably reduced preceding an IDH episode.

Literature around intradialytic SO_2_ measurement is more limited. ScvO_2_ has been shown to reduce with increasing UFV^27^^,^^28^ and to associate with survival,^29^ whereas variability in SaO_2_ and ScvO_2_ is predictive of IDH occurrence.^30^ Our results demonstrate that ScvO_2_ and eUBBF, but not SaO_2_, are notably reduced preceding an IDH event. This could be explained by the fact that ScvO_2_ and eUBBF (strongly related to ScvO_2_) can indicate decreased cardiac output and subsequent inadequate tissue oxygenation, unlike SaO_2_, which is more reflective of pulmonary function.^28^^,^^31^

These data may help to refine how CLM-measured RBV and SO_2_ are utilized and to better define outcomes to help build the evidence base necessary for widespread adoption.

### Associations between time of IDH onset and clinical variables

Patients prone to IDH in general were more likely to be female and comorbid, and have lower blood pressure, higher BMI, longer dialysis vintage, and higher IDWG (Table 1), which is consistent with previous literature.^32^ Most factors associated with IDH occurrence per se were associated with early-onset IDH, with the exceptions being higher BMI and IDWG and lower DCa, which were associated with IDH in general while being associated with late-onset IDH.

BMI is known to have complex associations with outcomes in kidney disease,^33^ and this is likely to extend to the relationship between BMI and IDH. BMI is linked to IDWG and therefore UFR, but patients with higher BMI appear to be able to better tolerate larger UFV and fluid depletion.^34^ Two studies showing that BMI had no association with IDH, whereas increased fat and reduced lean tissue were associated with IDH,^35^^,^^36^ may point to the problems in BMI as a surrogate for body composition. IDWG is independently linked to IDH^37^ and mortality^38^; however, IDWG is also strongly linked to nutrition, and this has been shown to impact on the relationship between IDWG and mortality.^39^ Body habitus and nutrition may be important factors in future work considering the timing of IDH.

Serum calcium is involved in myocardial contraction and therefore DCa may have a role in maintenance of blood pressure during HD.^40^ Clinical trials have demonstrated increasing DCa improves blood pressure but not symptoms associated with IDH^41^ and that profiling DCa leads reduces intradialytic blood pressure instability.^42^ The risk of indication bias with our data must be considered as prescription of DCa may be related to patients being prone to IDH. This may explain the association with early-onset IDH (Table 2) and the increase in both higher (3 mEq/L) and lower (2.25 mEq/L) prescriptions in sessions where IDH occurred (Table 1).

The association between UFR and mortality is well described,^43^ but there are surprisingly little data describing associations between UFR and IDH, although Dialysis Outcomes and Practice Patterns Study (DOPPS) data showed UFR >10 ml/h per kg is associated with greater odds of IDH.^44^ We found UFR to be lower in patients prone to IDH and increasing UFR to be associated with less chance of early-onset IDH. This could be related to normalization of UFR to body weight (given BMI was higher in IDH-prone patients) or potentially to the reduction of UFR prescriptions in unstable patients.

### Survival analysis

Kaplan-Meier analysis (Figure 5) and Cox analysis suggest that early-onset IDH is associated with both all-cause and cardiovascular mortality. Comorbidities, including chronic heart failure and autonomic dysfunction, which compromise the ability to compensate for fluid removal on dialysis, may be linked to early-onset IDH and the association with survival. Nevertheless, the associations were maintained after adjustment for a limited number of potential confounders (Table 3).

### Sensitivity analysis

Sensitivity analyses demonstrate the importance of how IDH is defined for both research and management of IDH. The addition of a minimal reduction in SBP to a nadir value, reducing the dependence on the initial SBP, predictably reduced the number of IDH incidents early in a session, although there was notable incidence in the first half of dialysis in all definitions used. We repeated all secondary analyses, including survival analysis, using the combined definition of a decline- and a nadir-based SBP in an attempt to reduce the imapct of IDH episodes relating to chronic hypotension rather than dialysis-induced hypotension. All results were robust to this alternative definition.

### Clinical implications

IDH is likely the result of several mechanistic pathways, although there is often little differentiation in treatment. Given the associations with mortality, intervening to prevent early-onset IDH may have significant impact on outcomes. Further research is needed to explore the mechanisms and potential interventions around IDH early in treatment, but promising options could include minimizing the effect of large osmotic changes in the early stages of dialysis (reduced blood flows and sodium modeling), preserving central blood volume (modified dialysate temperature, positional changes, and ultrafiltration profiles), and further investigation of the early dialysis-induced hypoxemia demonstrated elsewhere^45^ and supported by this study.

### Strengths and limitations

This is the first study aiming to characterize the time of onset of IDH. It is based on a large cohort from the network of Renal Research Institute centers across the United States. We included all patients who dialyzed in these clinics during the study period, removing selection bias. Sex, dialysis vintage, age, and race data are all comparable to US Renal Data System data sets, although comorbidity and antihypertensive medication data appear to be low, likely due to missing data being given the same status as a null response in the source database. IDH rates per session for the SBP-based definitions are also extremely similar to previously reported data,^17^ providing further support for the generalizability of the results. CLM data were not available for all patients, but the use of CLM is a unit level decision rather than based on patient need.

Consistent with observational data, residual confounding must be considered. Associations between modifiable variables, such as HD prescriptions, and IDH are likely to include confounding by indication.

### Conclusion

We have demonstrated a broad distribution in the time of onset of IDH throughout a treatment session, which is associated with clinical, treatment, and outcome variables, including survival. Consideration of time of IDH alongside well-described underlying mechanisms could potentially help to differentiate IDH episodes and facilitate effective use of interventions, something that remains a challenge.

## Disclosure

PK and ST hold stock in Fresenius Medical Care. JW is employed by Fresenius Medical Care North America. JGR, HZ, ST, and PK are employees of the Renal Research Institute. The Renal Research Institute is a wholly owned subsidiary of Fresenius Medical Care. All the other authors declared no competing interests.
