# Supplementary material for: The time of onset of intradialytic hypotension during a hemodialysis session associates with clinical parameters and mortality
Source: Kidney Int. 2021 Jun;99(6):1408–17. doi: 10.1016/j.kint.2021.01.018 (PMC8165353; doi:10.1016/j.kint.2021.01.018)
Supplement: Supplementary File (PDF) [file mmc1.pdf]

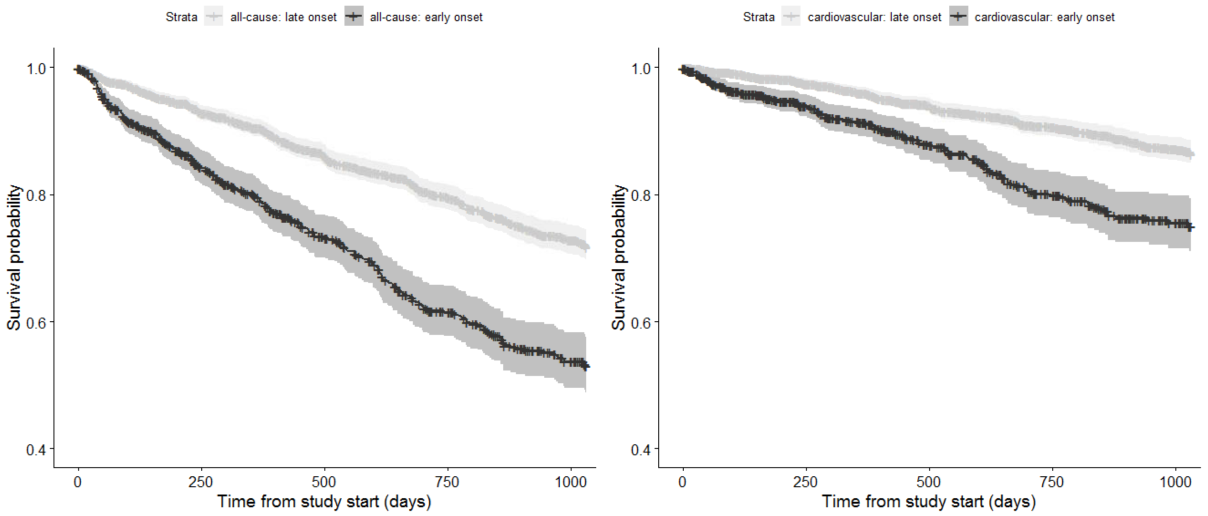

Supplementary figure S1: Sensitivity analysis: definition of IDH of SBP<90mmHg and a decline in SBP>30mmHg from pre-dialysis levels. Kaplan-Meier curves for patients who tended to have IDH in the first half of a session compared with those who tended to have IDH in the second half of a session for all-cause (left) and cardiovascular (right) mortality.

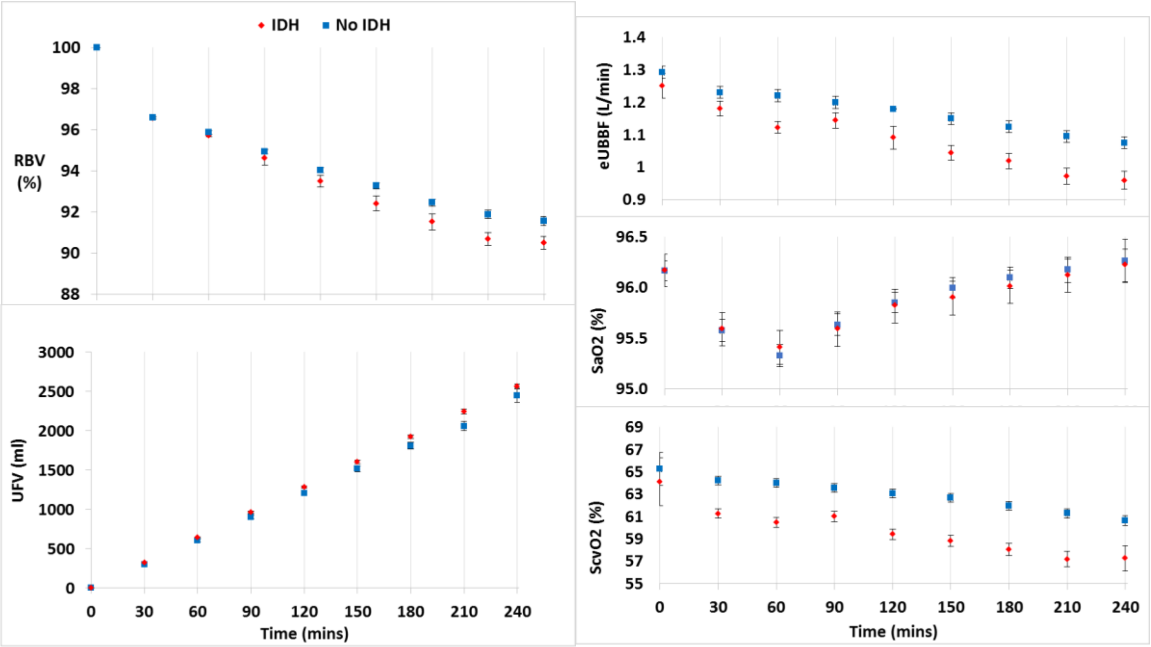

Supplementary figure S2: Sensitivity analysis: definition of IDH of SBP<90mmHg and a decline in SBP>30mmHg from pre-dialysis levels. Intradialytic relative blood volume (RBV), ultrafiltration volume (UFV), arterial oxygen saturation, (SaO_2), central venous oxygen saturation ($ScvO_2$) and estimated upper body blood flow (eUBBF). Each data point represents an average value at the start of every session-interval, stratified by whether IDH occurred in the subsequent 30 minutes or not, with error bars representing 95% confidence intervals

	Univariate		Multivariate (model 1)		Multivariate (model 2)	
**Variable**	**Odds ratio**	**95% CI**	**Odds ratio**	**95% CI**	**Odds ratio**	**95% CI**
IDH Prone	1.45	1.28-1.64*	1.83	1.22-1.54*	1.29	1.08-1.55*
Age (years)	1.02	1.02-1.02*	1.02	1.01-1.02*	1.02	1.01-1.02*
Gender Male	0.78	0.72-0.85*	0.76	0.70-0.82*	0.75	0.65-0.88*
BMI (kg/m^2)	0.98	0.98-0.99*	0.99	0.9-0.99*	0.98	0.97-0.99*
CHF	1.15	1.02-1.30*	1.11	0.99-1.26	1.15	0.93-1.42
Diabetes	0.96	0.88-1.05	0.99	0.90-1.07	1.07	0.91-1.26
PAD-PVD	1.04	0.88-1.23	0.90	0.77-1.06	0.74	0.56-0.99*
IDWG (kg)	0.93	0.92-0.94*	0.94	0.94-0.96*	0.91	0.86-0.96*
Ultrafiltration rate (ml/hr/kg)	0.98	0.98-0.99*	0.99	0.99-1.00*	1.00	0.98-1.01
Pre SBP (mmHg)	0.99	0.99-0.99*	0.98	0.99-0.99*	0.99	0.99-0.99*
Dialysis vintage (years)	1.00	0.99-1.01	1.00	0.99-1.01	1.00	0.99-1.02
Dialysate Calcium (mEq/L)	1.02	0.91-1.13	1.19	1.03-1.28*	1.43	1.09-1.87*
Albumin	0.61	0.55-0.67*	-	-	0.96	0.79-1.18
Dialysate to serum Na gradient (mEq/L)	1.00	0.98-1.02	-	-	1.00	0.98-1.02

Supplementary table S1: Mixed effects logistic regression for the odds of IDH occurring in the first half of a session compared to later in the treatment. IDH defined as SBP<90mmHg and a decline in SBP>30mmHg. Adjusted analysis for model 1 include all covariates available for all treatments (785682 sessions), whereas for model 2 includes all variables from model one plus albumin and sodium gradient in a limited number of treatments due to availability of these data (15994 sessions).
** indicates p<0.05.*

Variable	All cause mortality				Cardiovascular mortality			
	Univariate		Multivariate		Univariate		Multivariate	
	OR	95% CI	OR	95% CI	OR	95% CI	OR	95% CI
Early onset IDH	2.04	1.75-2.37*	1.43	1.19-1.71*	2.12	1.69-2.67*	1.58	1.21-2.07*
IDH prone	2.04	1.64-2.55*	1.61	1.25-2.08*	1.79	1.26-2.55*	1.62	1.10-2.39*
Age (years)	1.04	1.04-1.05*	1.03	1.03-1.04*	1.03	1.02-1.04*	1.02	1.01-1.03*
Dialysis vintage (years)	1.00	1.00-1.00	1.00	1.00-1.00	1.00	1.00-1.00	1.00	1.00-1.00
Gender Male	0.97	0.83-1.12	1.27	1.07-1.51*	1.04	0.83-1.30	1.29	0.99-1.67
Race (white vs. non white)	1.65	1.43-1.92*	1.48	1.25-1.76*	1.19	0.95-1.49	1.15	0.89-1.49
BMI (kg/m^2)	0.97	0.96-0.98*	0.97	0.96-0.99*	0.97	0.95-0.99*	0.97	0.95-0.99*
CHF	1.26	1.03-1.55*	1.18	0.95-1.46	1.43	1.07-1.93*	1.35	0.99-1.85
Diabetes	1.04	0.89-1.23	0.89	0.74-1.06	1.07	0.84-1.37	0.92	0.71-1.21
Albumin (g/dL)	0.26	0.23-0.21*	0.27	0.22-0.33	0.34	0.27-0.44*	0.39	0.28-0.54*
IDWG (kg)	0.94	0.91-0.96*	0.99	0.96-1.02	0.95	0.91-1.00	1.00	0.96-1.04

*Supplementary Table S2: Cox proportional hazards analysis of the association of early onset IDH with both all-cause and cardiovascular mortality. IDH defined as SBP<90mmHg and a decline in SBP>30mmHg. * indicates p<0.05.*
